# A comprehensive analysis of digital health-focused Living Labs: innovative approaches to dementia

**DOI:** 10.3389/fmed.2024.1418612

**Published:** 2024-07-10

**Authors:** Teodora Figueiredo, Luís Midão, Joana Carrilho, Diogo Videira Henriques, Sara Alves, Natália Duarte, Maria João Bessa, José María Fidalgo, Maria García, David Facal, Alba Felpete, Iván Rarís Filgueira, Juan Carlos Bernárdez, Maxi Rodríguez, Elísio Costa

**Affiliations:** ^1^CINTESIS@RISE, Biochemistry Lab of the Faculty of Pharmacy of the University of Porto, Porto, Portugal; ^2^Faculty of Pharmacy, Department of Biological Sciences, University of Porto, Porto, Portugal; ^3^Porto4Ageing - Competences Centre on Active and Healthy Ageing, Faculty of Pharmacy, University of Porto, Porto, Portugal; ^4^Santa Casa da Misericórdia de Riba D’Ave/CIDIFAD – Centro de Investigação, Diagnóstico, Formação e Acompanhamento das Demências, Braga, Portugal; ^5^CINTESIS@RISE, Instituto de Ciências Biomédicas Abel Salazar of the University of Porto, Porto, Portugal; ^6^UPTEC-Science and Technology Park of University of Porto, Porto, Portugal; ^7^ACIS-Agencia Gallega para la Gestión del Conocimiento en Salud, Santiago de Compostela, Spain; ^8^Department of Developmental Psychology, IDIS, University of Santiago de Compostela, Santiago de Compostela, Spain; ^9^AFAGA Alzheimer - Asociación de Familiares de Enfermos de Alzheimer y otras Demencias de Galicia, Vigo, Spain

**Keywords:** Living Labs, open innovation, digital health, dementia, innovation ecosystems

## Abstract

The increasing prevalence of dementia demands innovative solutions; however, existing technological products often lack tailored support for individuals living with this condition. The Living Lab approach, as a collaborative innovation method, holds promise in addressing this issue by actively involving end-users in the design and development of solutions adapted to their needs. Despite this potential, the approach still faces challenges due to its lack of recognition as a research methodology and its absence of tailored guidelines, particularly in dementia care, prompting inquiries into its effectiveness. This narrative review aims to fill this gap by identifying and analysing digital health Living Labs focusing on dementia solutions. Additionally, it proposes guidelines for enhancing their operations, ensuring sustainability, scalability, and greater impact on dementia care. Fifteen Living Labs were identified and analyzed. Based on trends, best practices, and literature, the guidelines emphasize user engagement, interdisciplinary collaboration, technological infrastructure, regulatory compliance, transparent innovation processes, impact measurement, sustainability, scalability, dissemination, and financial management. Implementing these guidelines can enhance the effectiveness and long-term impact of Living Labs in dementia care, fostering new collaborations globally.

## 1 Introduction

Among the challenges associated with the ageing population, dementia presents an increasingly pressing societal issue. Being one of the most prevalent neurodegenerative diseases with no cure currently available, dementia ranks at the top among the leading causes of disability and dependency among older people worldwide (1). In 2020 the global number of people living with dementia was estimated at over 55 million and it is expected to reach 139 million by 2050 (2). The caregiving burden is predominantly shouldered by informal carers, typically family members and friends of those living with dementia. As the prevalence of dementia continues to rise without effective treatment, and the cost of dementia care increases, the urgent need for alternative solutions becomes more apparent. This leads to a growing reliance on innovative technologies or services to provide new responses to those affected by dementia (3, 4).

In recent years, research on using technology for dementia has gained more attention. The main areas of technological development include diagnosis, assessment and monitoring, maintenance of function, leisure activities, and caregiving and management (5). Digital health strategies for people with dementia or cognitive impairment are diverse, including Artificial Intelligence (AI), Big Data platforms, and telemedicine for monitoring cognitive functions; Extended Reality – Virtual Reality (VR), Augmented Reality (AR) and Mixed Reality (MR) – for education, training, and treatment; and robots and smart home technologies to enhance daily activities and social skills (6).

Although various methods and approaches for designing technology exist, a considerable number of products currently available on the market are not tailored to meet the needs of persons living with dementia (7). Given this high rate of failure, it became imperative to actively involve end-users in co-creation processes, increasing relevance and attention directed toward the Living Lab approach (8, 9). Involving and engaging individuals living with dementia in these processes poses significant challenges due to their impaired cognitive abilities. Nevertheless, excluding them will cause difficulty in implementation in real-life scenarios and will probably decrease the hypothesis of success and acceptance of such solutions (10).

Although there is not a widely recognized definition of a Living Lab, this concept is centred on two main ideas: the real-life experimentation environment and the active involvement of users in the innovation process (11, 12). Operating across diverse contexts, Living Labs serve as dynamic spaces for testing, validating, developing, and co-creating throughout the entire design and commercialization process. They function as collective hubs for innovation, offering valuable insights, serving as testbeds for pioneering products, services, systems, and solutions, and helping to create a sense of community across the development process (11). Living Labs are a collaboration between multiple stakeholders. Four key groups of stakeholders are responsible for the successful implementation and development of a Living Lab: governmental bodies, industry, academic institutions, and end-users (quadruple helix approach) (13).

Recently, these collaborations have been transformed and innovation has been accelerated due to the emergence of Smart Cities, the Internet of Things (IoT), AI, ER, and Big Data paradigms, among others. These technological advancements have not only facilitated rapid access to innovation but also enabled transitions toward greater sustainability. Moreover, they have significantly enhanced the exchange of data and knowledge, serving as drivers for policy development and the scale-up of initiatives (14).

Since 2015, there has been a substantial increase in publications focusing on Living Labs. In the field of dementia, the total number is lower but the tendency to increase since 2015 is also found. Currently, there is a large number of actively functioning Living Labs on a global scale, with a particularly pronounced prevalence in European regions (12, 15). This approach has been frequently applied to the development of health devices, addressing mostly issues associated with vulnerable groups, such as older people and age-related diseases (16).

Publications addressing the diverse needs and expectations of people living with dementia, along with corresponding solutions, have emerged in the last few years but remained notably limited (17). A scoping review conducted in 2021 investigated Living Labs studies that focused on cognitive impairment and dementia-related solutions. The Living Labs identified were dedicated to enhancing the health, quality of life, independent living, home care, and safety of older adults with cognitive disorders or dementia. Additionally, they aimed to provide support for professional and family caregivers while alleviating their burdens (17). In the context of dementia Living Labs, technological products or services that support people to live independently and well at home, such as assistive technology, are the most common (18).

Despite the potential of the Living Lab approach and the successful development of products, services and solutions (19, 20), this methodology still faces several challenges. One significant issue is that Living Labs are usually unrecognized as a research methodology and, consequently, lack the credibility required for securing traditional research funding (21). Additionally, there is a lack of tailored and specific guidelines for Living Labs, particularly in the field of dementia.

To address the gap in research focusing on the distinct features and practices of Living Labs dedicated to dementia, and to meet the societal need for tailored digital health technologies for individuals affected by this condition, this narrative review aimed to identify and analyze the characteristics of digital health Living Labs with solutions for dementia. Thus, to answer the question “**What are the main characteristics of digital health Living Labs focused on dementia?**,” Living Labs with this focus were screened and analyzed. Insights into their collaborative ecosystems, user engagement approaches, technological infrastructure, regulatory compliance, innovation processes, impact on healthcare outcomes, and strategies for funding and resource management were collected. The findings of this research contributed to the formulation of a comprehensive set of guidelines intended to inform about the operation and development of future Living Labs in the field. By optimising the effectiveness and impact of forthcoming Living Labs, this initiative strives to enhance approaches to develop digital health technology tailored to dementia care.

In 2015, an attempt to propose a Living Lab protocol for evaluating interventions in the context of dementia was already undertaken, albeit limited to three study cases featuring specific interventions and a restricted participant pool. The main findings from this study underscore the importance of actively involving relevant stakeholders from the inception of the process. Moreover, it stated that the industry stakeholders’ needs should be aligned with the Living Lab’s needs to gather usable insights for their interventions (22). Another study explored academic-practice partnerships of the Living Lab approach to dementia care and concluded that researchers should take the initiative in shaping collaborations and providing opportunities for stakeholder engagement (23). Recent research delved into the operational aspects of Living Labs incorporating real products from Small and Medium-sized Enterprises (SMEs) in the everyday living environment of individuals living with dementia. The study emphasized the need for diverse stakeholder compositions and expertise. Furthermore, Living Lab researchers were identified as pivotal connectors and buffers between individuals living with dementia and SMEs, facilitating the adoption of technological products (18). It also highlighted that the implications of living with dementia need to be acknowledged and respected by care professionals, researchers and companies which may imply the adaptation of the technology, methodologies, or evaluation process, requiring time, flexibility, patience and commitment by all of the institutions involved (18).

## 2 Materials and methods

The process of selecting Living Labs involved the application of multiple screening methods. Initially, four electronic databases (PubMed, Web of Science, Scopus, EBSCOhost) were searched to identify articles referencing Living Labs specifically dedicated to dementia and/or cognitive impairment. It is important to note that, because dementia is typically diagnosed when cognitive impairment becomes severe enough to affect social or occupational functioning (24), the study included Living Labs focused on dementia, cognitive impairment, and both.

The search strings and outcomes are detailed in the Supplementary Table S1. Following the removal of duplicates and non-English written articles, a pool of 57 full-text articles was screened for Living Labs focused on digital health, with solutions on dementia and/or cognitive impairment. From these databases, 23 articles mentioned established Living Labs and 7 Living Labs were identified with the desired focus.

Complementary to this, a search of the most established global Living Labs network was undertaken to identify other Living Labs with the intended focus, the European Network of Living Labs (ENoLL). ENoLL was chosen due to its international presence and extensive network (25). The search was performed in February 2024. From this screening method, 5 additional Living Labs with the intended focus were retrieved.

Furthermore, web searches were conducted to uncover additional relevant Living Labs. This retrieved 3 additional Living Labs. A total of 15 Living Labs were analyzed. Information about these Living Labs was gathered from their official websites and relevant scientific publications, including original research articles, reports, and case studies.

In the analysis of these Living Labs, each Living Lab was analyzed considering the following aspects: (1) type of living lab, (2) collaborative ecosystem, (3) user-centric approach, (4) technological infrastructure, (5) regulatory and ethical compliance, (6) innovation processes and methodologies, (7) impact and success metrics, (8) sustainability and scalability, (9) knowledge sharing and dissemination, (10) funding and resource management. These aspects were chosen based on ENoLL evaluation criteria for Living Labs eligibility (26).

Regarding the type of Living Lab, three distinct types were considered: research-driven Living Lab, Living testbed, and Living Lab as a service. To clarify, a research-driven Living Lab is characterized by a primary focus on scientific investigation and experimentation. This type of Living Lab prioritises academic research and collaboration with research institutions. Their primary goal is to generate new knowledge and advance scientific understanding. Living testbeds are environments specifically designed for the practical testing and validation of technologies, solutions or innovations. These testbeds aim to replicate real-world conditions to assess the feasibility, performance, and functionality of new concepts. Living Lab as a service refers to a model where organizations offer Living Lab facilities and expertise as a service to external entities, such as businesses, startups, or government agencies. This approach allows external partners to leverage the infrastructure, resources, and knowledge of an established Living Lab without having to develop and maintain their own. It is pertinent to note that certain Living Labs may fall into more than one of these designated categories (27).

Then, collaborative ecosystems were assessed aiming to explore whether Living Labs led collaborative initiatives and projects with other entities such as universities, industry, healthcare providers, government agencies and others.

Concerning the user-centric approach, the focus shifted to examining the integration of end-users in co-creating and evaluating digital health solutions, along with exploring the methods and tools employed to gather feedback and ideas from these users. In this context, it is essential to distinguish between two key concepts: co-creation and co-design. Co-creation involves a collaborative approach to creative problem-solving that engages diverse stakeholders throughout all stages of an initiative, encompassing problem identification, solution generation, implementation, and evaluation. On the other hand, co-design is a subset of co-creation, specifically emphasising the active collaboration among stakeholders in designing solutions tailored to a pre-defined problem (28).

Turning to technological infrastructure, the analysis centred on studying the availability of the necessary infrastructure for testing and validating digital health products. This also encompassed an examination of the integration of emerging technologies such as AI, IoT, VR, wearables, etc. in testing processes.

Subsequently, regulatory and ethical compliance was considered, particularly focusing on the adherence to frameworks related to health, digital health, and data protection and security in the healthcare field.

Concerning innovation processes and methodologies, the analysis encompassed the transparency and structure of the innovation process (prototyping, testing, and scaling up). This also involved evaluating the utilization of design thinking, agile methodologies, or other relevant approaches.

The impact and success metrics of Living Labs were analyzed with a focus on their demonstration of improving healthcare outcomes, efficiency, and patient experiences, accompanied by clear success metrics and evidence of achieved results.

This was followed by exploring the sustainability and scalability plans and initiatives of the Living Labs, which included strategies for integrating successful solutions into health systems.

In terms of knowledge sharing and dissemination, emphasis was placed on the efforts of Living Labs to share knowledge, best practices, and lessons learned with the wider community through dissemination activities.

Lastly, funding and resource management were considered, exploring budgetary allocations, funding sources, as well as the effective utilization and management of resources to sustain the operations and objectives of Living Labs.

## 3 Results

The data collection methodology allowed the identification of 15 Living Labs (Table 1). The majority of the Living Labs selected were European (*n* = 11): France (*n* = 4), England (*n* = 2), Spain (*n* = 2), Germany (*n* = 1), Scotland (*n* = 1) and Sweden (*n* = 1). Living Labs from Canada (*n* = 2), Australia (*n* = 1), and the United States of America (*n* = 1) were also included.

**Table 1:** Identified Living focused on digital health, with solutions for dementia and/or cognitive impairment (*n* = 15), along with their corresponding countries, as well as a brief overview outlining the purpose and objectives of each Living Lab.

Living Lab	Country	General description and objectives of the Living Lab
LUSAGE Gerontechnology Living Lab	France	The LUSAGE Gerontechnology Living Lab specialises in designing and providing assistive technology for older adults, focusing on enhancing their autonomy and quality of life, particularly those living with cognitive impairment (e.g., Mild Cognitive impairment, Alzheimer’s disease and related dementias), and supporting their informal and formal caregivers.
Bremen Ambient Assisted Living Lab (BAALL)	Germany	At the BAALL, new ambient assisted living technology is tested for usability in a 60 m^2^ apartment designed for two people. This apartment includes standard living areas and follows the design-for-all principle. This Living Lab anticipates the scenarios that may arise from age-related physical or cognitive impairments and plans to compensate for them using technological assistance.
The Living Lab at Liverpool John Moores (LJMU)	England	The LJMU collaborates with people living with dementia to develop innovative solutions for their daily challenges. The team works with the business sector, academia, and service providers, focusing on co-creating memory-enabling technologies for the health and social care of people living with dementia.
Laval-ROSA Transilab	Canada	The Laval-ROSA Transilab uses Living Lab and learning health system approaches. It aims to improve care transitions between different settings - Family Medicine Groups, home care, and community services -, ultimately improving the care of people living with dementia and their caregivers.
Médéric Alzheimer Foundation Living Lab	France	The Médéric Alzheimer Foundation Living Lab develops and evaluates products, services, and interventions for people living with dementia, involving them throughout the process. It aims to enhance the integration and quality of life for older adults with Alzheimer’s and related illnesses. Its main focus is assessing the impact of psychosocial interventions, such as cognitive stimulation through technology use, art therapy, music therapy, and reminiscence, on the quality of life for those living with Alzheimer’s disease.
DOMUS (Laboratoire de Domotique et informatique Mobile à l’Université de Sherbrooke)	Canada	The DOMUS features a versatile infrastructure for designing, implementing, and evaluating cognitive orthotics (assistive technology) that supports various activities of daily living (ADLs), to help people with cognitive impairments - Alzheimer’s disease, mental retardation, schizophrenia, or traumatic brain injury – to live independently. DOMUS operates three Living Lab variants: a smart apartment for short-term studies; a housing unit enabling long-term studies in a technology-rich real house; and mobile setups for long-term studies in older adults’ homes.
Swinburne Living Lab	Australia	The Swinburne Living Lab aims to increase the quality of life and independence of vulnerable user groups, including older adults, individuals living with dementia, those with disabilities and culturally diverse groups. This Living Lab plays a key role in the development of Assistive Robots for the future of healthcare. Their goal is to create innovative solutions that are easily embraced by users because they fit with their actual needs.
MINDLab	Spain	The MINDLab aims to enhance social healthcare and promote independent living among older individuals and those facing autonomy challenges, such as people living with dementia, through innovative solutions. This Living Lab focuses on older adult’s home settings. Its activities range from assessment of needs and co-design to implementation in simulated Living Lab environments and real home pilots, with a thorough analysis of usability challenges. Companies have the opportunity to test their technology in real environments.
Idea	Spain	The Idea Living Lab aims to improve the quality of life of older people, including individuals with cognitive impairments. It provides services and products in the field of care and digitalization. The Idea Living Lab also provides services to public administrations, private entities, and technology companies, including gerontological consulting, research partnerships, product viability analysis, co-design and testing of ICT products.
Pasteur Innovative Living Lab of Nice	France	The Pasteur Innovative Living Lab of Nice fosters the emergence and growth of digital technologies in the domain of homecare and independent living. This Living Lab is equipped with a model apartment that is designed as a showcase and a testing platform for technologies supporting independent living and autonomy.
Lab4Living	England	The Lab4Living aims to address real-world issues that impact health and well-being, developing products, services and interventions that promote dignity and enhance quality of life. Established to promote user-driven innovation through co-creation, Lab4Living focuses on various projects, with a particular emphasis on researching ageing and age-related diseases such as dementia.
StrathLab	Scotland	The StrathLab aims to translate health and care innovation into equitable and accessible care for all. Its focus is on improving socially inclusive and sustainable care at home through technology. StrathLab is connected to a set of networks such as Carer and Dementia Networks. StrathLab has innovation facilities including VR labs and simulations of real-world environments.
The Technology and Aging Lab at McLean Hospital	United States	The Technology and Aging Lab at McLean Hospital provides an environment for optimising treatments and providing support for patient-centred research initiatives. This Living Lab researches the influence of digital tools on psychiatric care throughout life, with a special emphasis on older adults and individuals living with dementia and their caregivers. The investigations cover digital diagnosis tools, technology-enhanced therapies, and the incorporation of technology into patient care processes.
Living Lab Vieillissement et Vulnérabilités (LL2V)	France	The LL2V is focused on testing, evaluating, researching, and developing prevention and support solutions for common vulnerabilities in older adults, including cognitive impairment. Its projects involve the creation of digital solutions such as innovative VR entertainment and the development of Integrated Technology Assistance for daily living, among others.
Digital Innovation for Dementia Care (DIDEC)	Sweden	The DIDEC aims to enhance innovation, competitiveness, and growth among SMEs focusing on technology for dementia care. It aims to achieve this through enhanced methodologies for collaborative and challenge-driven innovation within dementia care. The initiative utilizes a dedicated testbed for its activities.

Among the 15 identified Living Labs, the primary research focus centred around leveraging digital technologies to improve/benefit: quality of life, well-being, dignity, cognition, autonomy, independent living, accessibility, social innovation, solutions focused on diagnosis, and the healthcare of people with dementia. Additionally, several Living Labs had solutions to reduce the burden on families, informal and professional caregivers and other health professionals of people living with dementia (Table 1).

The products tested/developed included assistive technologies (e.g., remote monitoring systems and context-aware applications), environmental assistance “smart homes” by intelligent appliances and furniture (e.g., kitchen appliances, refrigerator and bed), intuitive user interfaces (e.g., TV and voice control), health monitoring technologies (e.g., apps), digital diagnostics and phenotyping, digital therapeutics and clinical implementation (e.g., sensing technology to assess behavioral and psychological symptoms and to monitor treatment response in people with dementia).

The characteristics of each Living Lab were collected, and the main findings are presented in Table 2. The main categories of the Living Labs studied were research-driven Living Lab (*n* = 12), Living testbed (*n* = 9) and Living Lab as a service (*n* = 3).

**Table 2:** Characteristics of the selected Living Labs (*n* = 15).

Criteria for evaluating Living Labs	Living Labs (*n* = 15)
**Type of Living Lab**	
Classification or categorization of Living Labs.	Research-driven Living Lab (*n* = 12) Living testbed (*n* = 9) Living lab as a service (*n* = 3) Information not available (*n* = 1)
**Collaborative ecosystems**	
Partnerships with different entities.	Yes (*n* = 12) Information not available (*n* = 3)
Collaborative initiatives and projects.	Yes (*n* = 12) Information not available (*n* = 3)
**User-centric approach**	
Integration of end-users in the co-creation and evaluation of digital health solutions.	Co-creation (*n* = 6) Co-design and user testing (*n* = 3) Only co-design (*n* = 2) Only user testing (*n* = 1) Co-learn, co-design and co-effectuate (*n* = 1) Information not available (*n* = 2)
Methods and tools for gathering user feedback and insights.	Yes (*n* = 12) Information not available (*n* = 3)
**Technological infrastructure**	
Availability of necessary technology infrastructure for testing and validating digital health products.	Yes (*n* = 9) Information not available (*n* = 6)
Integration of emerging technologies (e.g., AI, IoT, wearables) in the testing process.	Yes (*n* = 6) Information not available (*n* = 9)
**Regulatory and ethical compliance**	
Adherence to regulatory frameworks and ethical guidelines related to healthcare and digital health.	Yes (*n* = 2) Information not available (*n* = 13)
**Data privacy and security**	
Robust data privacy and security measures to protect sensitive health-related data.	Yes (*n* = 0) Information not available (*n* = 15)
**Innovation process and methodologies**	
Transparent and structured innovation process, including ideation, prototyping, testing, and scaling.	Yes (*n* = 10) Information not available (*n* = 5)
**Impact and success metrics**	
Demonstrated impact on improving healthcare outcomes, efficiency, or patient experiences.	Yes (*n* = 10) Information not available (*n* = 5)
Clear success metrics and evidence of achieved outcomes.	Yes (*n* = 6) Information not available (*n* = 9)
**Sustainability and scalability**	
Plans for sustainability and scalability of the Living Lab and its initiatives.	Yes (*n* = 1) Information not available (*n* = 14)
Strategies for integrating successful solutions into mainstream healthcare systems.	Yes (*n* = 1) Information not available (*n* = 14)
**Knowledge sharing and dissemination**	
Efforts to share knowledge, best practices, and lessons learned with the broader community.	Yes (*n* = 14) Information not available (*n* = 1)
**Funding and resource management**	
Adequate funding sources and efficient management of financial resources.	Yes (*n* = 4) Information not available (*n* = 11)
Allocation of resources for research, development, and operations.	Information not available (*n* = 15)

Regarding the collaborative ecosystem, the majority of the Living Labs analyzed are known to carry out or are carrying out partnerships with different entities (*n* = 12), including industry, startups, SMEs or larger companies, R&D organizations or centres, universities, healthcare providers and civic sectors and associations, building projects and various collaborative initiatives. However, only a small number (*n* = 2) reported having partnerships with policy-makers and representatives of ethical committees. For example, the LUSAGE Gerontechnology Living Lab demonstrated a comprehensive engagement across a wide spectrum of stakeholders, including in their network, policy-makers, health insurers, representatives of ethical committees and other relevant stakeholders (29). It’s important to acknowledge that available information was limited in this field for the remaining Living Labs, preventing definitive conclusions regarding their partnership structure.

Based on the available information, within the selected Living Labs, most have included co-creation with the end-users (*n* = 6), others include co-design and user testing (*n* = 3), only co-design (*n* = 2), or only user testing (*n* = 1). Interestingly, the DIDEC Living Lab follows a co-learn, co-design and co-effectuate pathway (20). For the co-creation and co-design and to gather feedback and insights from users, several strategies were reported, including focus groups, interviews, direct observations, surveys, questionnaires (e.g., pre-and post-intervention), workshops, meetings or sessions, mapping and strategic foresight.

Concerning technological infrastructure, a significant number reported having the necessary mechanisms to guarantee effective testing and adequate validation of the results of their products, services or interventions (*n* = 9). Some had fully equipped simulated real environments. For instance, the Bremen Ambient Assisted Living Lab (BAALL) features all standard living areas—bedroom, bathroom, dressing area, living and dining room, kitchenette, and home office —within a 60 m2 apartment, suitable for accommodating two people on a trial basis (30). Similarly, the LUSAGE Gerontechnology Living Lab boasts a versatile architectural layout that can be tailored to conduct *in-situ* observations, mimicking a home-like setting, according to the requirements of each project. This setup offers a controlled environment for studying user interactions with technological devices via non-intrusive methods such as an eye tracker, different types of sensors, and an audio and video recording system (29). Interestingly, the StrathLab uses VR to model ‘real-world’ environments such as pharmacies, or various spaces within a household (31). Alternatively, one Living Lab identified relied on external institutions for assessments in real-life conditions, for instance, hospital departments, day-care centres or residential establishments for dependent older adults. Related to this, some Living Labs reported integrating emerging technologies in testing processes (*n* = 6), using mostly different types of wearables and sensors, but also, AI, RV and AR.

Regarding regulatory and ethical compliance, as well as data protection and security, there was limited information accessible online. Only two Living Labs explicitly state compliance with regulatory frameworks and ethical guidelines. The Living Lab at Liverpool John Moores University emphasized the importance of ethical considerations, ensuring that individuals deemed too vulnerable or lacking capacity should be identified and should not participate. As part of their methodology, they also provide individuals living with dementia the option to have another person present during interviews, whether it be their informal caregiver or formal carer, as a supportive measure (21).

In terms of the innovation process carried out and the methodologies applied, some of the selected Living Labs lacked publicly available information about their innovation process. Nevertheless, many exhibit transparent and structured innovation processes, including ideation, prototyping, testing and scaling up, primarily employing problem-solving methodologies (*n* = 10). Based on the information available, the most predominant is design thinking, i.e., human-centred design to tackle problem-solving needs; only a small number of Living Labs utilize agile methodology, i.e., an iterative and incremental process that is beneficial in uncertain contexts (32).

In terms of impact and success metrics of the selected Living Labs, the majority demonstrate their impact on improving healthcare outcomes, efficiency, or patient experiences (*n* = 10). This is evidenced through the sharing of success stories, the introduction of products and interventions in the market and the publication of scientific articles, case studies or reports. However, fewer have clear and available success metrics and evidence of achieved outcomes (*n* = 6).

Concerning sustainability and scalability, the absence of information prevented a detailed analysis of the Living Labs’ plans, initiatives, or strategies in this domain. Only one Living Lab has available information about this. As a result, it is not feasible to examine how these entities aim to integrate successful solutions into conventional healthcare systems or their broader sustainability and scalability efforts. It is also important to highlight that some of them exhibit lower maturity or are relatively recent, with a temporal scope constrained within the bounds of specific research projects. The only exception is the Laval-ROSA Transilab which has clear plans for sustainability, beyond the planned project funding. For instance, they intend to employ a research agent to facilitate coordination and foster internal sustainability (33). Additionally, this Living Lab also aims to support the learning transfer from Transilab to other health organizations (33).

Regarding sharing and dissemination, almost all the Living Labs reported efforts to share knowledge, best practices and lessons learned with the broader community (*n* = 14). Additionally, half of the Living Labs analyzed are members of ENoLL (*n* = 7). ENoLL, a global network of open Living Labs, plays a crucial role in this dissemination by fostering a dynamic, multi-layered innovation ecosystem that facilitates cooperation and synergy among its members and external stakeholders (25). Besides ENoLL, the Swinburne Living Lab is also a member of the Australian Living Lab Innovation Network (ALLiN) (34). Furthermore, the dissemination of knowledge by some of the identified Living Labs is promoted through the publication of editorials, literature reviews, case studies, book reports and other scientific articles, training, workshops, congresses, webinars, newsletters and/or posters (20, 21, 29, 30, 33, 35–38).

Finally, regarding financing and resource management, the larger part of the Living Labs provides limited or no information on this aspect. From our analysis, only four Living Labs have some information about financial support. For the majority, project funds are described as the main source of budgetary support. The importance of financial support was particularly stressed by the LUSAGE Gerontechnology Living Lab which underscored the need for a sustainable business model. This model should address key issues such as defining roles for private (such as banks and insurance companies) and public stakeholders, recognizing the value of innovative solutions, and establishing legal and political frameworks for sustainability strategies (29).

## 4 Discussion

Many digital health solutions for dementia do not meet the specific needs, expectations and capabilities of individuals (39). This highlights the importance of creating customized technology and the need for the Living Lab approach, which involves end-users in the development process through a collaborative multidisciplinary network. While this approach is gaining increased interest from researchers and policymakers as a “practical innovation ecosystem,” there remains a significant gap in understanding its operation and resultant outcomes, prompting inquiries into its effectiveness (40).

With this in mind, the present study focuses on examining Living Labs that utilize digital solutions for individuals living with dementia or cognitive impairment. It aims to analyze their main characteristics to ultimately develop guidelines and highlight best practices for future initiatives in this area, and potentially aid in harmonising procedures regarding the operation of Living Labs in the field of dementia. To achieve this, 15 Living Labs were identified and analyzed, and several aspects came into consideration.

It is important to note that ENoLL already has a list of 20 indicators of the success of the performance of a Living Lab that can be seen as guidelines to follow. These indicators are based on the following areas: active user involvement, multi-method approach, multi-stakeholder participation, orchestration, real-life setting, and co-creation (26). There are other tools, similar to this one, that have been developed mostly in European projects [e.g., SISCODE Self-assessment questionnaire by Schmittinger et al. (41, 42) but are still in the testing phase or are not easily accessible due to scattered publications (41–43). Although these indicators are critical, they are general and lack the specificity needed for the operation and development of digital health Living Labs in dementia care.

The main focus of the Living Labs identified was to improve the quality of life and health of people living with dementia. However, it is worth mentioning that certain Living Labs prioritised the needs and designed solutions that targeted not only people with dementia but also individuals in their ecosystems, including caregivers, family members, and health professionals. Given the escalating demand for family caregivers due to the ageing population and the growing prevalence of dementia, there is a pressing need for tools that alleviate their burdens (physical, psychological and financial). These caregivers, who are predominantly older individuals themselves, require assistance and support in managing their caregiving responsibilities, enhancing their understanding (e.g., disease, care tasks, legal issues), and accessing healthcare services (44). Moreover, there is a noticeable willingness among caregivers to adopt new technologies to aid in their caregiving tasks (44).

Interdisciplinary collaboration also emerged as a crucial aspect of the selected Living Labs, promoting cooperation among researchers, healthcare professionals, technology experts, designers and people living with dementia to leverage diverse perspectives and expertise in solution development (18). However, it is fundamental to cultivate strategic partnerships with policymakers and ethical committees to ensure the sustainability of Living Lab initiatives. Ensuring long-term engagement with users and stakeholders is highlighted as essential, emphasising continuous feedback gathering, impact assessment, and adaptation to evolving user needs (29).

A significant hurdle faced by Living Labs in this field stems from the recognition that unique challenges arise in the process of co-creating products, services, and practices with people living with dementia. Communicating with designers and articulating their thoughts in a traditional co-design setting proves to be challenging for people with dementia (45). However, this design-driven approach to Living Labs has already proven effective in improving the value proposition of an innovative technological solution in the context of dementia care (46).

Within the studied Living Labs, most included co-creation with the end-users, while others included co-design and/or user testing. In this setup, users may either be seen as passive subjects to be observed or can actively participate as equal co-creators, offering valuable insights into the development of sustainable products and services. It is essential to emphasize that within a Living Lab approach, users should be regarded as partners in the innovation process, rather than just subjects of study (47). The selected Living Labs used different strategies to gather feedback and co-create with their end-users, such as focus groups, interviews, surveys, workshops, and strategic foresight exercises. While there is no standard practice in the literature, common methods for involving people with dementia in all phases of development include interviews and observations (48).

It is important to note that these approaches differ in the nature and intensity of the relationship between designers and users. A systematic review of involving people living with dementia in developing supportive technologies highlighted a lack of specific knowledge about the research methods and materials required to actively engage these individuals throughout the development process. It suggests that successful co-design with people living with dementia may not yet exist or is unpublished. The review found that the people involved were typically in mild to moderate stages of the condition. In all the studies reviewed, the initial idea for the technology or service had already been formed before including people with dementia. None of the articles measured whether the participants felt like equal partners in the process (48).

Co-creation with people with dementia can require multiple moments of explaining and repeating instructions, methodologies may need to be adapted to improve accessibility and timeframes may need extending (18). It is important to highlight that, although core symptoms such as reduced retrospective and abstract thinking, the course of dementia can vary, both between and within individuals, in an unpredictable way (48, 49). This is reflected in how they interact with and adopt technology (50). Therefore, designers and researchers should focus on the individual’s current abilities when using or testing technology (50). Despite these challenges, individuals living with dementia often exhibit a sense of purpose and curiosity toward testing new products, which fosters their willingness to participate in such initiatives (18). Additionally, support from informal and formal caregivers can enhance the ability of people living with dementia to use the technology (50). Usually, caregivers also play a vital role in explaining and stimulating the use of technologies, which implies that the caregivers also need to embrace the technological product or service and see the value it adds to their daily care practice (51).

The selected Living Labs exhibit some gaps and weaknesses that may impede their overall effectiveness and long-term impact. One significant limitation is the lack of transparent communication channels and overall information about the Living Lab, which may hinder openness toward new partners, collaborations, investors and public visibility and interaction. This also extends to critical information about regulatory frameworks, ethical guidelines, data privacy and security measures, funding sources, and efficient financial management for research, development, and operations.

The lack of solutions to integrate the existing healthcare system may also hinder adoption and interoperability. A recent review showed that the Living Lab approach contributes to the successful implementation of innovations in healthcare. It also reported that for this successful implementation, it is necessary six factors: early involvement of end-users, appropriate timing, effective leadership, openness to change, sense of ownership and organizational support (52).

Additionally, some of the Living Labs have a short-term duration, confined to the duration of specific research projects, which raises concerns about sustainability. The ability to continue project activities after the project concludes is jeopardized, potentially limiting the lasting impact these initiatives could have. Consequently, valuable effort, expertise, and knowledge acquired during these projects are at risk of being lost. Additionally, Living Labs frequently exhibit localized and small-scale scopes, presenting difficulties in achieving scalability. To attract larger-scale innovative enterprises, Living Labs must collaborate at national and international levels, overcoming this scalability challenge (53).

Another issue is that several of the identified Living Labs do not seem to undertake project evaluations or assess their impact. This lack of systematic evaluation hampers the progress of Living Labs, as it becomes challenging to learn from experiences and enhance future endeavors (54). Although Living Labs are beginning to pay attention to sharing their outcomes and benefits, only a few have focused on evaluating or measuring their performance (12).

Finally, the lack of a higher number of published articles or other dissemination activities restricts the broader accessibility of valuable insights and best practices in this field. Addressing these gaps is crucial for fostering the growth, sustainability, and impact of a Living Lab. In fact, questions about the effectiveness and outcomes of Living Lab initiatives are partly owed to the paucity of published evidence and insufficient reports of performance evaluations (40).

### 4.1 Guidelines for digital health Living Labs focused on dementia

Drawing from trends, best practices, and limitations observed in the analyzed Living Labs in this narrative review, as well as insights from existing literature discussed above, a comprehensive set of guidelines is proposed for Living Labs employing digital solutions for individuals living with dementia or cognitive impairment. These guidelines encompass 10 pivotal areas (Table 3).

**Table 3:** Guidelines proposed for the operation and development of digital health Living Labs focused on dementia.

Enhancing collaborative ecosystems	Stress the necessity of fostering interdisciplinary collaboration among researchers, healthcare professionals, technology experts, designers, and caregivers to ensure holistic solution development. Encourage strategic partnerships with policy-makers, ethical committees, advocacy organizations, and community groups to promote the sustainability and scalability of Living Lab initiatives.
Establishing a user-centric approach	Integrate end-users, including people with dementia, caregivers, and healthcare professionals, in the co-creation process from the outset. This involvement should extend beyond mere consultation to active collaboration Emphasize the importance of co-creation and co-design methodologies tailored to the unique needs and challenges faced by individuals living with dementia. These methodologies should accommodate various cognitive abilities and communication styles, facilitating active participation and meaningful engagement throughout all stages of innovation. Advocate for the development of user-friendly and accessible communication channels and methodologies to facilitate the involvement of individuals with varying degrees of cognitive impairment. This may involve employing multiple modalities such as visual aids, simplified language, and interactive tools to facilitate understanding and engagement.
Technological infrastructure and emerging technologies	Ensure the availability of well-equipped simulated environments for effective testing and validation of digital health products. Advocate for adaptable and inclusive technological infrastructure to accommodate the diverse needs and preferences of individuals living with dementia Embrace emerging technologies such as VR, AR, AI, IoT, wearables, and robotics in testing processes.
Regulatory and ethical compliance	Stress the critical need for adherence to regulatory frameworks related to health and digital health and ethical guidelines, particularly regarding data privacy and security measures, to protect sensitive health-related data of individuals living with dementia. Emphasize transparent communication of regulatory compliance measures and ethical considerations to stakeholders and the broader community to build trust and foster accountability.
Transparent innovation processes and methodologies	Implement transparent and structured innovation processes, incorporating ideation, prototyping, testing, and scaling up. Utilize design thinking and agile methodologies methods to enhance innovation processes. Ensure inclusive decision-making by providing opportunities for stakeholders to contribute to the decision-making process and clearly outline how decisions are made. Maintain accessible and well-documented records of the innovation processes and methodologies employed and make resources, protocols, and methodologies easily available to all involved parties.
Demonstrating impact and success metrics	Establish clear success metrics for outcomes, efficiency (e.g., cost–benefit analysis, product/solution adoption rates), and patient experiences (e.g., user feedback and satisfaction). Regularly assess and report the impact of Living Lab initiatives on improving the quality of life and/or health of people living with dementia.
Sustainability and scalability planning	Develop sustainability and scalability plans and initiatives, outlining strategies for integrating successful solutions into conventional healthcare systems. Foster long-term partnerships and collaborations to ensure the continued success and growth of Living Lab initiatives. Encourage Living Labs to develop long-term sustainability and scalability plans beyond the duration of specific research projects, leveraging strategic partnerships and diversified funding options. Advocate for collaboration at national and international levels to overcome scalability challenges and attract larger-scale innovative enterprises, ensuring the broader adoption of successful solutions.
Knowledge sharing and dissemination	Establish open and accessible communication channels to facilitate the sharing of knowledge among Living Lab stakeholders and the broader community. Stress the importance of publishing articles and engaging in dissemination activities to increase the accessibility of valuable insights and best practices in the field. Encourage active participation in collaborative networks and platforms to facilitate knowledge exchange and project partnerships, leveraging existing networks such as ENoLL and similar organizations.
Financial and resource management	Highlight the necessity of transparent financial structures and efficient management of funding sources, addressing key issues such as defining roles for private and public stakeholders. Advocate for diversified funding options and strategies to mitigate financial risks in innovation projects, ensuring the sustainability and longevity of Living Lab initiatives.
Continuous monitoring and improvement	Implement a robust monitoring system to track the progress of Living Lab initiatives. Regularly review and update the action plan based on the evolving technological, regulatory, and healthcare landscape, i.e., iterative evaluation. Regularly benchmark and analyze outcomes against successful models to pinpoint areas for improvement and adapt Living Lab strategy in response to evolving goals, emerging trends, and the dynamic nature of innovation.

These pivotal areas include the establishment of collaborative ecosystems, promoting interdisciplinary engagement among dementia researchers, healthcare practitioners, technologists, and caregivers. Additionally, a user-centric approach, where individuals living with dementia are engaged throughout all stages of innovation, is prioritized and tailored to the specific cognitive and functional intricacies of these individuals. Ensuring a robust technological infrastructure is essential, finely tuned to address the unique needs and challenges inherent in dementia care. Adhering to regulatory and ethical standards is emphasized to safeguard the integrity and privacy of sensitive health data. Transparent innovation processes are advocated, requiring clear documentation of methodologies and decisions throughout the innovation lifecycle. Moreover, the guidelines stress the importance of demonstrating impact through measurable success metrics, as well as planning for sustainability and scalability, and facilitating knowledge sharing and dissemination. Efficient financial and resource management is highlighted, alongside the implementation of continuous monitoring and improvement mechanisms, allowing for iterative refinement and adaptation of strategies in response to evolving technological landscapes and user needs within the dementia care paradigm.

By addressing these areas, Living Labs can create a comprehensive environment for developing digital health solutions tailored to the specific needs of individuals living with dementia. These guidelines, designed to be actionable, empower Living Labs to tackle challenges and leverage best practices, fostering sustainable innovation through interdisciplinary collaboration, active end-user involvement, and strategic partnerships. Furthermore, they offer a framework for continuous improvement, ensuring adaptability to evolving technologies and user needs. By adhering to these guidelines, the Living Lab community can elevate the quality and impact of their initiatives, ultimately enhancing health outcomes and quality of life for people living with dementia. These guidelines provide practical recommendations for researchers, policymakers, and other stakeholders interested in advancing innovation in this field.

### 4.2 Limitations of the narrative review

This study has some limitations that need to be addressed. The process of selecting Living Labs may have introduced bias, as it relied on the publication of scientific articles and networks. This approach may have overlooked relevant Living Labs that are not mentioned in published scientific articles or belong to ENoLL, however, to overcome this, additional web searches were carried out. Additionally, the majority of the identified Living Labs were from European countries, with fewer from other regions. This geographic imbalance may limit the generalizability of the findings, as different regions may have unique healthcare systems, regulatory frameworks, and cultural factors influencing Living Lab operations. Finally, the analysis of Living Labs relied on publicly available information from official websites and scientific publications. However, the completeness and accuracy of this information may vary, leading to potential gaps or inaccuracies in the assessment of Living Lab characteristics and activities.

## 5 Conclusion

The rise of dementia within an ageing population demands innovative solutions, with Living Labs offering promising avenues for co-creating and testing interventions. In this study, 15 digital health Living Labs focused on dementia and/or cognitive impairment were examined and guidelines for the operation and development of these Living Labs were constructed. Key findings reveal the importance of user engagement and interdisciplinary collaboration. Challenges include integration in the healthcare system, communication gaps, limited scalability, and lack of systematic evaluation. These challenges underscore the need for a holistic approach to address the multifaceted issues hindering the effectiveness and long-term impact of Living Labs, an approach that holds promise as a practical innovation ecosystem. Proposed guidelines emphasize user-centric approaches for people living with dementia, specific collaborative ecosystems, technological infrastructure, regulatory compliance, transparent innovation processes, impact measurement, sustainability planning, knowledge sharing, financial management, and continuous improvement. Implementing these guidelines can enhance the effectiveness and long-term impact of Living Labs in dementia care. Moreover, the guidelines suggested have the potential to serve as a valuable resource for Living Labs, focusing on similar solutions, on a global level. This will pave the way for new and successful collaborations.

## Author contributions

TF: Data curation, Formal analysis, Investigation, Methodology, Validation, Visualization, Writing – original draft. LM: Conceptualization, Data curation, Methodology, Visualization, Writing – review & editing. JoC: Conceptualization, Data curation, Methodology, Visualization, Writing – review & editing. DV: Conceptualization, Data curation, Methodology, Visualization, Writing – review & editing. SA: Conceptualization, Writing – review & editing. ND: Conceptualization, Writing – review & editing. MB: Conceptualization, Writing – review & editing. JF: Conceptualization, Writing – review & editing. MG: Conceptualization, Writing – review & editing. DF: Conceptualization, Writing – review & editing. AF: Conceptualization, Writing – review & editing. IF: Conceptualization, Writing – review & editing. JuC: Conceptualization, Writing – review & editing. MR: Conceptualization, Writing – review & editing. EC: Conceptualization, Funding acquisition, Project administration, Supervision, Writing – review & editing.
