# Supplementary material for: A comprehensive analysis of digital health-focused Living Labs: innovative approaches to dementia
Source: Front Med (Lausanne). 2024 Jul 10;11:1418612. doi: 10.3389/fmed.2024.1418612 (PMC11266068; doi:10.3389/fmed.2024.1418612)
Supplement: Supplementary file 1 [file Table_1.docx]

**Supplementary Table 1.** Overview of the search strings and the number of articles retrieved per database. The search was performed with no specific restriction on study design, setting, or date. The search was performed on 5^th^ February 2024.

| **Database** | **Search strings** | **Results (number of articles retrieved, n)** |
| --- | --- | --- |
| **PubMed** | "living lab*"[Title/Abstract] AND "dement*"[Title/Abstract] OR "amentia*"[Title/Abstract] OR "cognitive impair*"[Title/Abstract] OR "cognitive defect"[Title/Abstract] OR "alzheimer*"[Title/Abstract] OR "amnesia"[Title/Abstract] OR "neurocognitive disorder*"[Title/Abstract] OR "cognition disorder*"[Title/Abstract] OR "traumatic psychose*"[Title/Abstract] OR "Korsakoff"[Title/Abstract] OR "Huntington"[Title/Abstract] OR "Lewy Body"[Title/Abstract] OR "delirium"[Title/Abstract] OR "aphasia*"[Title/Abstract] OR "apraxia*"[Title/Abstract]) | n=17 |
| **Web of Science** | ((TS=(“living lab*”)) AND TS=(Dement* OR Amentia* OR cognitive impair* OR “cognitive defect" OR Alzheimer* OR amnesia OR neurocognitive disorder* OR cognition disorder* OR traumatic psychose* OR Korsakoff OR Huntington OR "Lewy Body" OR delirium OR Aphasia* OR Apraxia* )) | n=33 |
| **Scopus** | TITLE-ABS-KEY ( ( "living lab*" ) AND ( dement* OR amentia* OR "cognitive impair*" OR "cognitive defect" OR alzheimer* OR amnesia OR "neurocognitive disorder*" OR "cognition disorder*" OR "traumatic psychose*" OR korsakoff OR huntington OR "Lewy Body" OR delirium ) | n=47 |
| **EBSCOhost** | AB “living lab*” AND AB ( Dement* OR Amentia* OR cognitive impair* OR “cognitive defect" OR Alzheimer* OR amnesia OR neurocognitive disorder* OR cognition disorder* OR traumatic psychose* OR Korsakoff OR Huntington OR "Lewy Body" OR delirium OR Aphasia* OR Apraxia* )  +  TI “living lab*” AND TI ( Dement* OR Amentia* OR cognitive impair* OR “cognitive defect" OR Alzheimer* OR amnesia OR neurocognitive disorder* OR cognition disorder* OR traumatic psychose* OR Korsakoff OR Huntington OR "Lewy Body" OR delirium OR Aphasia* OR Apraxia* ) | n=16 (11+5) |
| Total | | n=113 |
| Total after removing duplicates and non-English written articles | | n=57 |
